# Supplementary material for: Nucleus accumbens core single cell ensembles bidirectionally respond to experienced versus observed aversive events
Source: Sci Rep. 2023 Dec 18;13:22602. doi: 10.1038/s41598-023-49686-x (PMC10730531; doi:10.1038/s41598-023-49686-x)
Supplement: Supplementary file 1 — Supplementary Figures. [file 41598_2023_49686_MOESM1_ESM.docx]

**
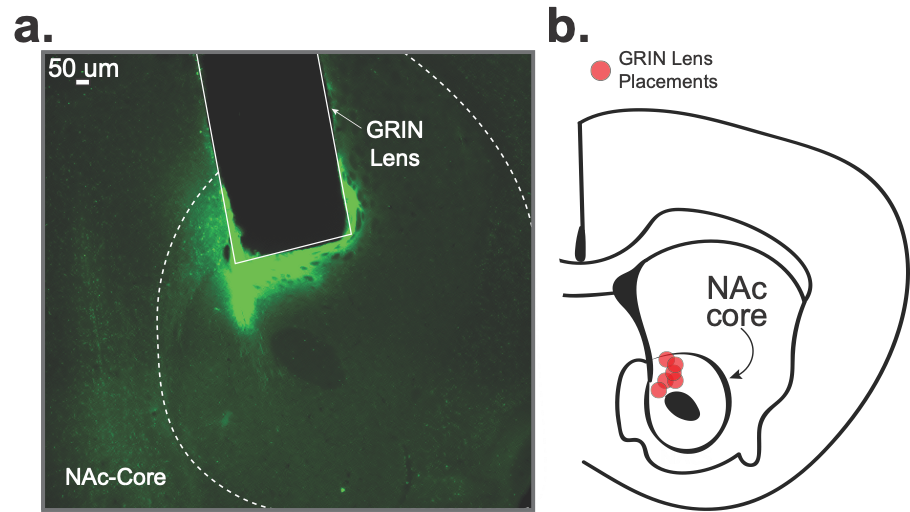
**

**Supp Figure 1. Representative histology. (a)** Representative image showing the expression of GCaMP in the NAc core and the placement of the GRIN lens relative to the viral expression. **(b)** Map showing GRIN lens placement in all mice.


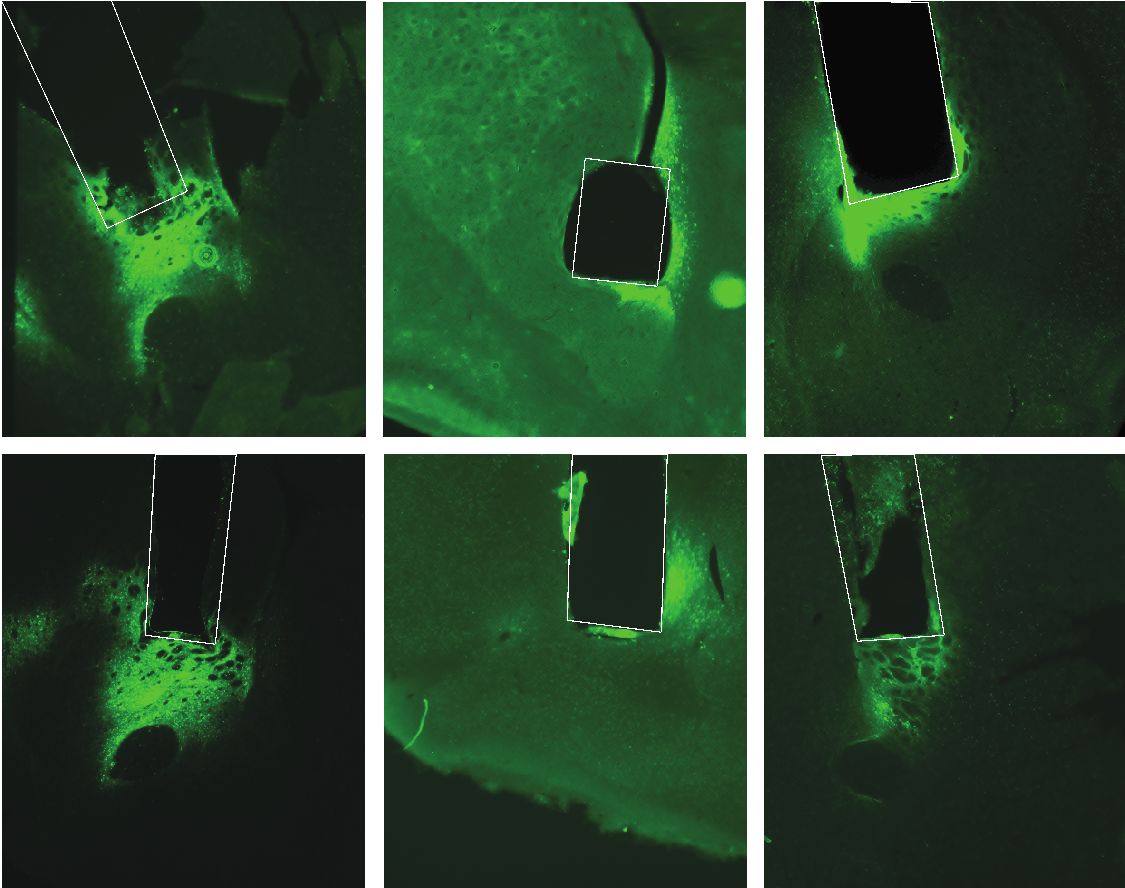


**Supp Figure 2. Representative histology images. (a)** Representative image showing the expression of GCaMP in the NAc core and the placement of the GRIN lens relative to the viral expression for each subject. **
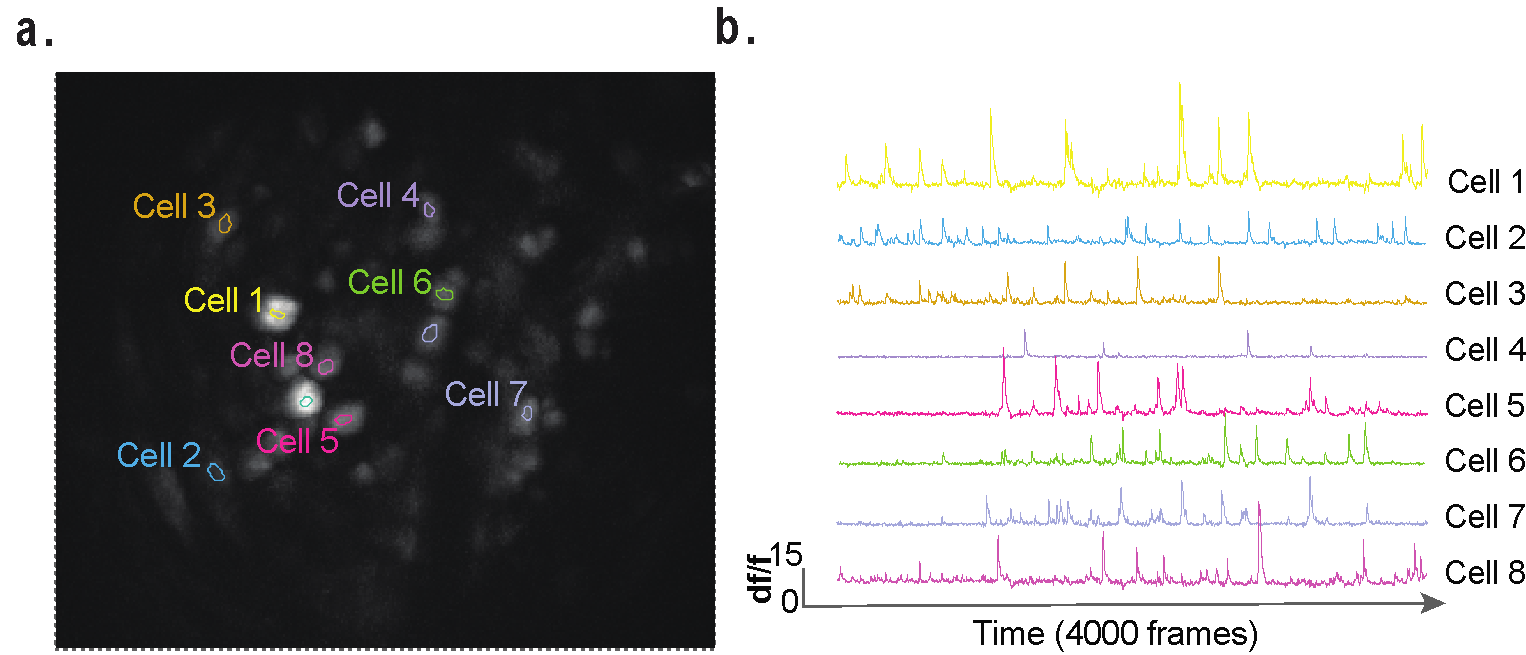
**

**Supp Figure 3. (a)** Representative cell map showing detected single cells from an individual animal. **(b)** Representative calcium traces from individual neurons identified in one animal.


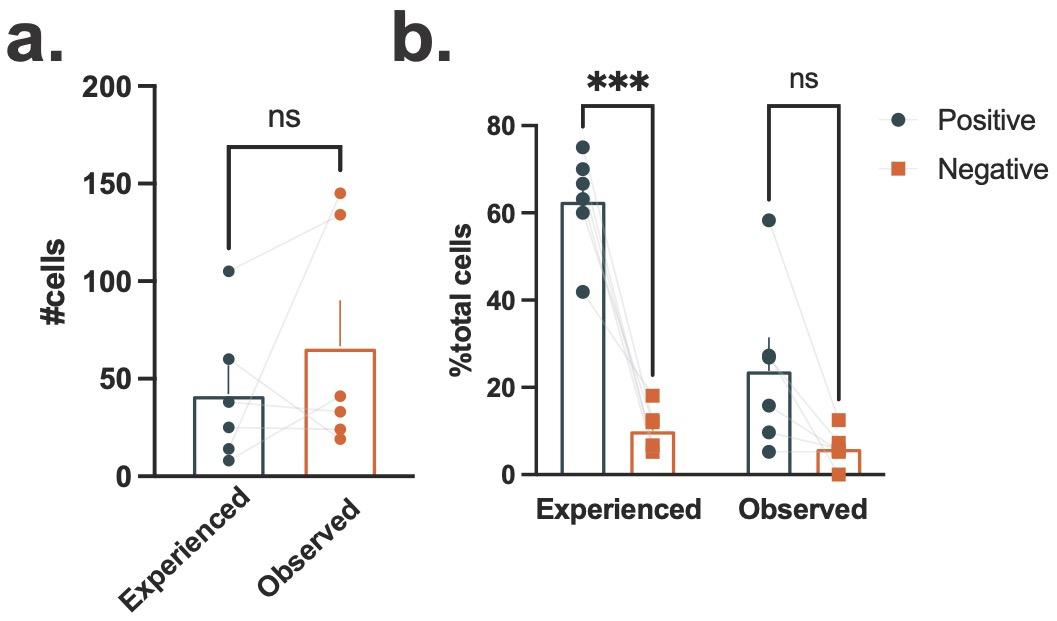


**Supp Figure 4. Total and positive/negative responsive cell numbers.** (**a**) The total number of detected cells from the “Experienced” and “Observed” footshock sessions did not differ significantly (paired t-test, *t*_5_=1.015, *p*=3567, n=6 mice). (**b**) The number of positive response cells was significantly larger than the number of negative response cells for the “Experienced Shock” session but these numbers did not significantly differ for the “Observed Shock” session (2-way ANOVA; Session x Response type interaction F_(1,5)_=15.14, p=0.0115, Sidak post-hocs for Experienced p=0.0008, for Observed p= 0.0736, n=6 mice). Data represented as mean ± S.E.M. *** *p* < 0.001, ns = not significant.


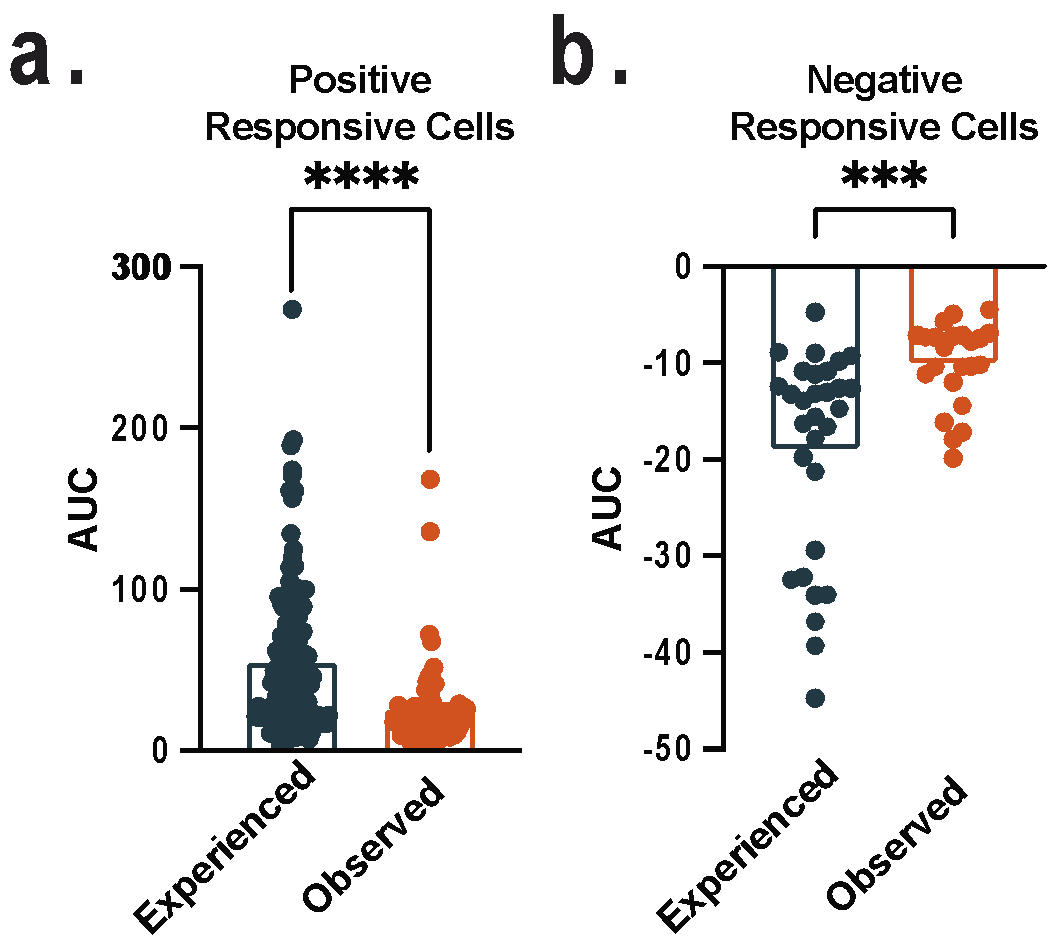


**Supp Figure 5. Single cell responses to the experienced footshocks were stronger compared to the responses to observed footshocks.** (**a**) Single cell responses from the cells that showed a significant positive response were significantly stronger during the experienced footshock session (unpaired t-test, *t*_209_=5.037, *p*<0.0001, n=71-140 cells). (**b**) Single cell responses from the cells that showed a significant negative response were significantly stronger during the experienced footshock session (unpaired t-test, *t*_51_=3.751, *p*=0.0005, n=23-30 cells). Data represented as mean ± S.E.M. *** *p* < 0.001, **** *p* < 0.0001.


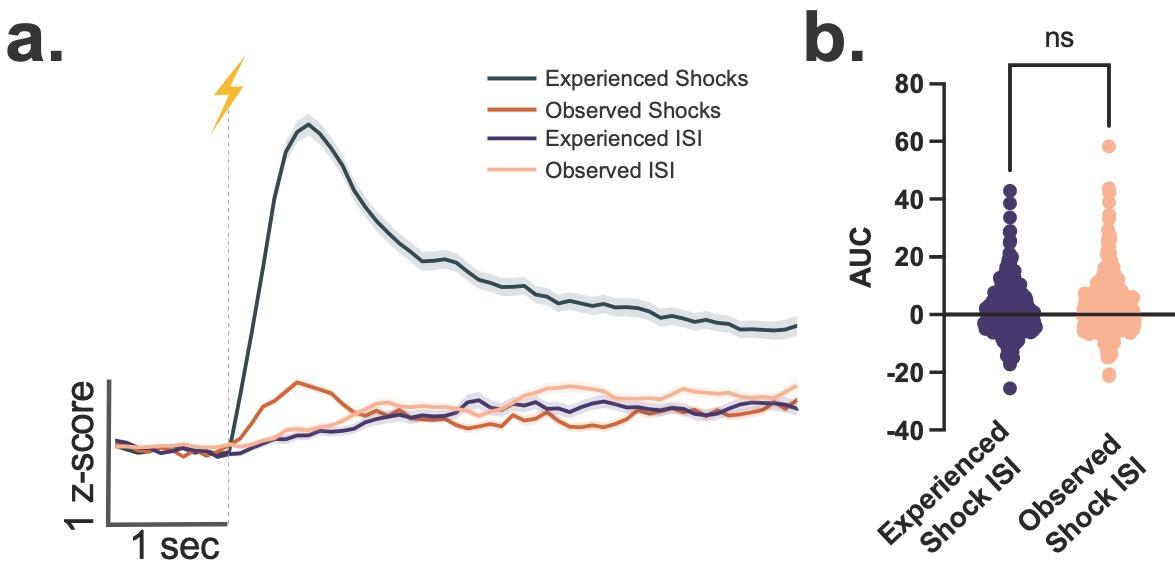


**Supp Figure 6. Baseline single cell responses during the inter-stimulus interval did not differ between Experienced and Observed footshocks.** (**a**) Mean cell response to the experienced versus observed footshock presentations as well as cell responses during the inter-stimulus interval (ISI) across all cells from all animals. (**b**) The mean area under the curve (AUC) for the population response did not differ between the Experienced and Observed footshock ISI periods (unpaired t-test, *t*_644_=1.587, *p*=0.1130, n=250-396 cells). Data represented as mean ± S.E.M., ns = not significant.


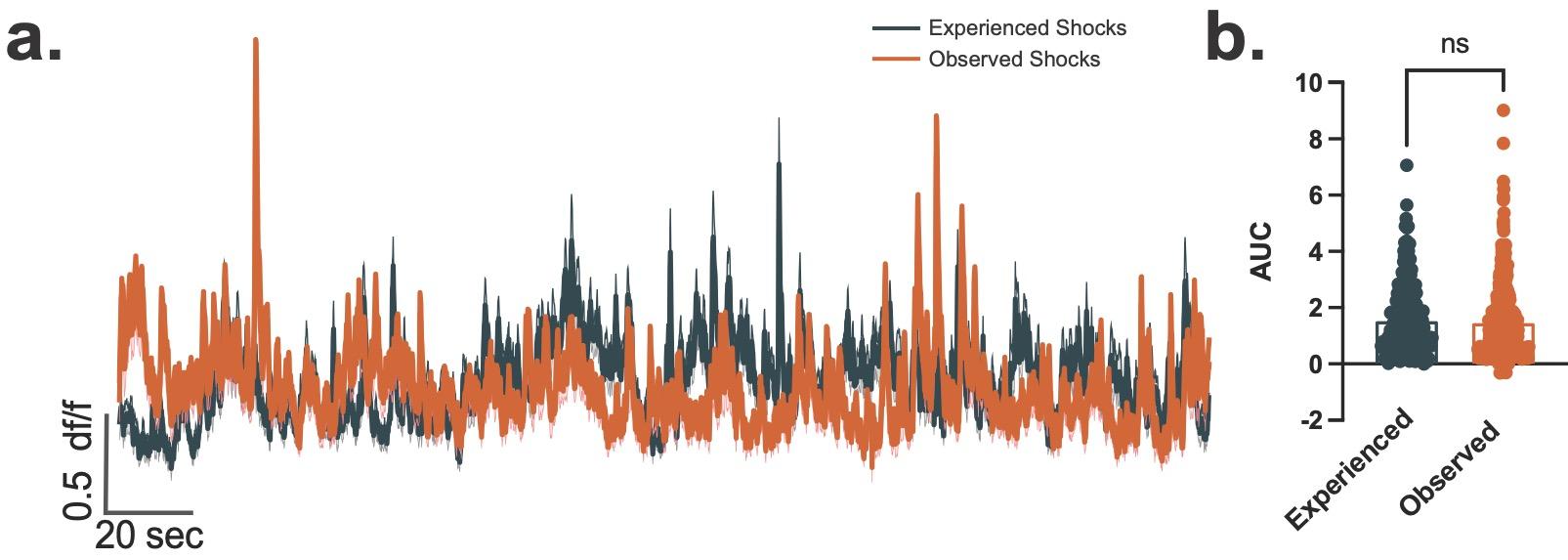


**Supp Figure 7. Baseline single cell calcium responses during the Experienced versus Observed footshocks sessions did not differ.** (**a**) Mean cell calcium response for the whole duration of the experienced versus observed footshock sessions. (**b**) Mean area under the curve (AUC) for the baseline population response did not differ between the Experienced and Observed footshock sessions (unpaired t-test, *t*_644_=0.6997, *p*=0.4843, n=250-396 cells). Data represented as mean ± S.E.M., ns = not significant.


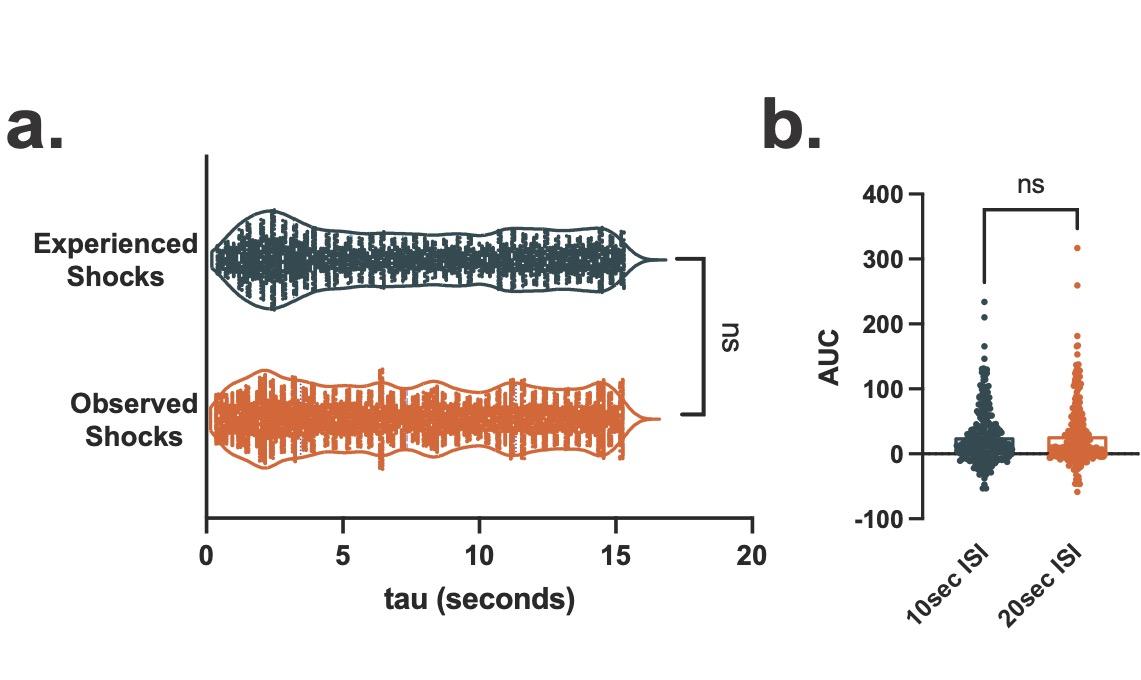
**Supp Figure 8. Time to decline to baseline did not differ after experienced versus observed footshocks.** (**a**) Time (seconds) for each cell shock response to decline to 1/3 of its peak magnitude (tai) did not differ between experienced and observed footshocks (unpaired t-test, *t*_13021_=0.4497, *p*=0.6529, n=4731-8292 responses). (b) The cell responses (area under the curve, AUC) to the experienced footshocks following 10 vs 20 second inter-stimulus interval (ISI) did not differ (paired t-test, *t*_249_=0.7505, *p*=0.4537, n=250 cells). Data represented as mean ± S.E.M., ns = not significant.


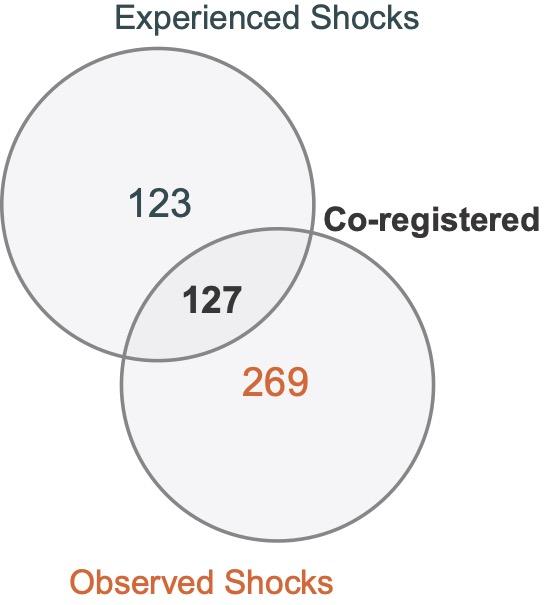


**Supp Figure 9. Experienced and observed footshock responsive cell ensembles showed a large overlap.** Of all cells that were detected during the experienced versus observed footshock sessions, there was a large number of cells that were detected in both sessions (n = 127 cells).


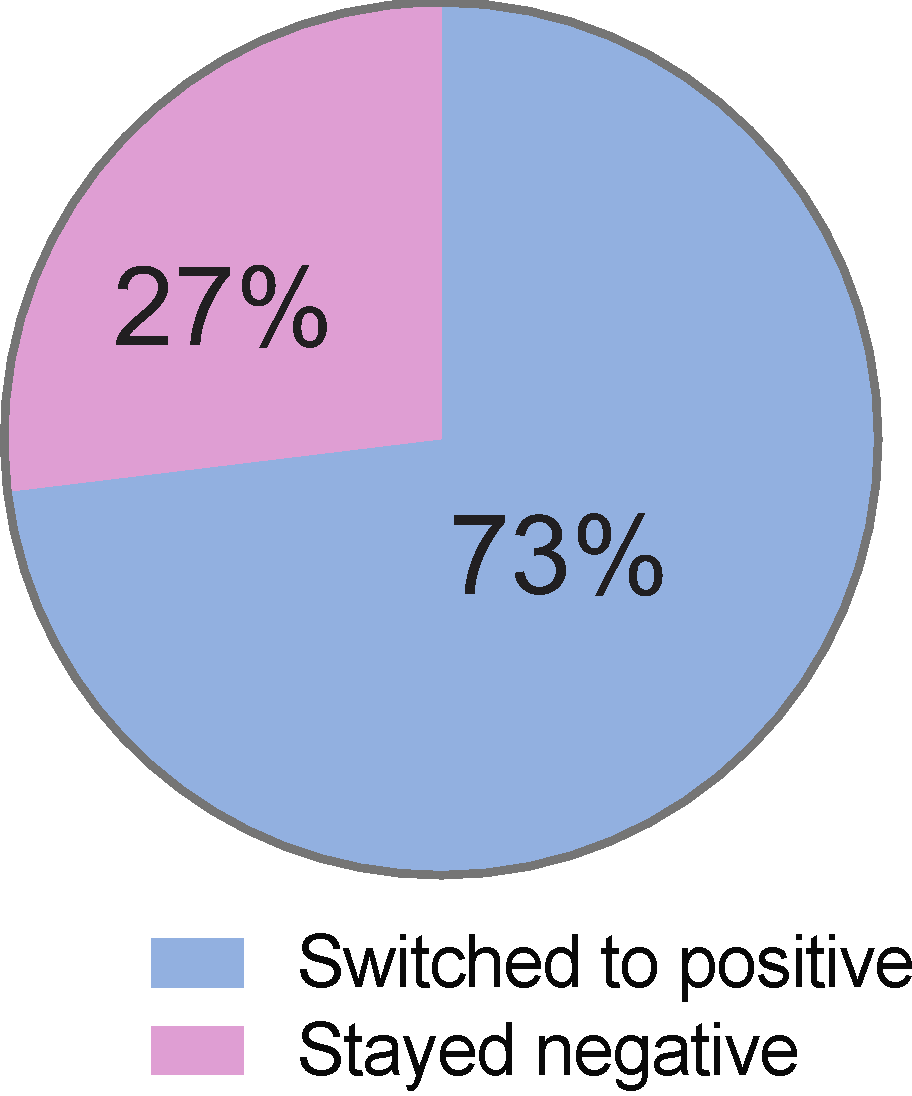


**Supp Figure 10.** The majority of the cells that showed a negative response to the experienced footshocks switched to a positive response when the mice observed another mouse receiving footshocks (73%).
